# Mitochondrial oxidative stress causes insulin resistance without disrupting oxidative phosphorylation

**DOI:** 10.1074/jbc.RA117.001254

**Published:** 2018-03-29

**Authors:** Daniel J. Fazakerley, Annabel Y. Minard, James R. Krycer, Kristen C. Thomas, Jacqueline Stöckli, Dylan. J. Harney, James G. Burchfield, Ghassan J. Maghzal, Stuart T. Caldwell, Richard C. Hartley, Roland Stocker, Michael P. Murphy, David E. James

**Affiliations:** From the ‡ Charles Perkins Centre, School of Life and Environmental Sciences, and; the ‡‡ Charles Perkins Centre, Sydney Medical School, University of Sydney, Camperdown, New South Wales 2006, Australia,; the § Vascular Biology Division, Victor Chang Cardiac Research Institute, Darlinghurst, New South Wales 2010, Australia,; ¶ St. Vincent's Clinical School, University of New South Wales, Sydney, New South Wales 2052, Australia,; the ‖ School of Chemistry, University of Glasgow, Glasgow G12 8QQ, United Kingdom, and; the ** MRC Mitochondrial Biology Unit, Hills Road, University of Cambridge, Cambridge CB2 0XY, United Kingdom

**Keywords:** superoxide ion, oxidative stress, hydrogen peroxide, mitochondria, insulin resistance, insulin, adipocyte, muscle, adipose tissue, Mitochondrial dysfunction

## Abstract

Mitochondrial oxidative stress, mitochondrial dysfunction, or both have been implicated in insulin resistance. However, disentangling the individual roles of these processes in insulin resistance has been difficult because they often occur in tandem, and tools that selectively increase oxidant production without impairing mitochondrial respiration have been lacking. Using the dimer/monomer status of peroxiredoxin isoforms as an indicator of compartmental hydrogen peroxide burden, we provide evidence that oxidative stress is localized to mitochondria in insulin-resistant 3T3-L1 adipocytes and adipose tissue from mice. To dissociate oxidative stress from impaired oxidative phosphorylation and study whether mitochondrial oxidative stress *per se* can cause insulin resistance, we used mitochondria-targeted paraquat (MitoPQ) to generate superoxide within mitochondria without directly disrupting the respiratory chain. At ≤10 μm, MitoPQ specifically increased mitochondrial superoxide and hydrogen peroxide without altering mitochondrial respiration in intact cells. Under these conditions, MitoPQ impaired insulin-stimulated glucose uptake and glucose transporter 4 (GLUT4) translocation to the plasma membrane in both adipocytes and myotubes. MitoPQ recapitulated many features of insulin resistance found in other experimental models, including increased oxidants in mitochondria but not cytosol; a more profound effect on glucose transport than on other insulin-regulated processes, such as protein synthesis and lipolysis; an absence of overt defects in insulin signaling; and defective insulin- but not AMP-activated protein kinase (AMPK)-regulated GLUT4 translocation. We conclude that elevated mitochondrial oxidants rapidly impair insulin-regulated GLUT4 translocation and significantly contribute to insulin resistance and that MitoPQ is an ideal tool for studying the link between mitochondrial oxidative stress and regulated GLUT4 trafficking.

## Introduction

Insulin lowers blood glucose, in part, by stimulating glucose uptake into adipose tissue and skeletal muscle. Insulin achieves this by increasing cell-surface levels of glucose transporter type 4 (GLUT4) via activation of the phosphatidylinositol 3-kinase/Akt signaling pathway. This process is defective in insulin resistance, a pathophysiological state that is a major risk factor for the development of type 2 diabetes and other metabolic diseases.

Insulin resistance in adipocytes and muscle has been reported to be caused by a number of systemic insults, including increased caloric intake, sedentary behavior, hyperinsulinemia, glucocorticoids, and inflammation. Although the relationship between these stresses remains unclear, many involve altered mitochondrial function, including incomplete fatty acid oxidation leading to the accumulation of lipid intermediates like acylcarnitines (1); increased mitochondrial oxidants (2–6); and dysregulated mitochondrial turnover, fission/fusion (7), and proteostasis (8–10). Further, other cellular stresses linked to insulin resistance, including ceramide accumulation, may also impair mitochondrial function (11), and insulin-sensitizing drugs, like metformin and berberine, mediate their beneficial effects by modulating mitochondrial function in a manner that remains incompletely understood (12, 13).

Many of these studies have shown a major role for mitochondria in the pathogenesis of insulin resistance. However, mitochondrial dysfunction *per se* appears unlikely to be the primary cause of insulin resistance. Whereas some genetic (14, 15) or pharmacologic (16) interventions that specifically compromise mitochondrial function have been shown to cause insulin resistance, other genetic interventions that impair mitochondrial function actually improve whole-body insulin action (17, 18). In contrast to defects in mitochondrial function, an increase in mitochondrial oxidants is a more consistent feature of insulin resistance *in vitro* (2, 4, 6) and *in vivo* (3, 5, 6) and in humans (3). Furthermore, genetic or pharmacologic interventions to detoxify superoxide radical anion (O_2_^˙̄^) or hydrogen peroxide (H_2_O_2_) specifically in mitochondria overcome insulin resistance (3–5). Whereas these studies imply that mitochondrial O_2_^˙̄^/H_2_O_2_ are intimately involved in the development of insulin resistance, it remains unclear whether scavenging such oxidants also affected mitochondrial function. For example, expression of mitochondria-targeted catalase in mice has been reported to improve insulin sensitivity via increased energy expenditure and fatty oxidation (19, 20). Thus, there is a pressing need to show a direct link between mitochondrial oxidants and insulin resistance independently of mitochondrial function.

Investigations into the effects of mitochondrial oxidants on insulin action have been hampered by a lack of tools to acutely and specifically induce mitochondrial oxidants without inhibiting the mitochondrial electron transport chain (ETC).^5^ Genetic approaches, such as deletion of mitochondrial superoxide dismutase (SOD2), elevate oxidants chronically, which may lead to compensatory responses (21). Small-molecule approaches to generate O_2_^˙̄^, such as paraquat and antimycin A, suffer from limitations such as redox cycling in compartments other than the mitochondria and inhibition of the ETC, respectively.

To circumvent these limitations, in the present study, we have used mitochondria-targeted paraquat (MitoPQ), which enriches in the mitochondrial matrix and generates O_2_^˙̄^ by redox cycling at the flavin site of complex I (22). MitoPQ was reported to increase mitochondrial O_2_^˙̄^ in cultured myoblasts, as assessed by increased hydroethidine-derived fluorescence, and elicited responses associated with increased mitochondrial O_2_^˙̄^, such as decreased cell viability (22). We found that MitoPQ induced mitochondrial O_2_^˙̄^ and H_2_O_2_ without impairing whole-cell oxygen consumption. MitoPQ acutely impaired insulin-stimulated GLUT4 translocation in both adipocytes and muscle cells. This occurred without impairment in proximal insulin signaling to Akt or TBC1D4. MitoPQ caused a specific defect in insulin regulation of GLUT4 trafficking, and other actions of insulin remained intact, as has been observed in other models of insulin resistance (23). Thus, treatment with MitoPQ enabled us to recapitulate previously observed features of insulin resistance. This demonstrates for the first time that an acute increase in mitochondrial oxidants, independent of mitochondrial dysfunction, is sufficient to induce insulin resistance. Our data in *in vitro* and *in vivo* models of insulin resistance indicate that oxidative stress in insulin-resistant adipocytes appears specific to mitochondria, and we propose that these mitochondrial oxidants induce a retrograde signaling pathway to impair insulin-regulated delivery of GLUT4 to the PM.

## Results

### Insulin resistance is associated with mitochondrial oxidative stress in adipocytes

**Figure 1.**
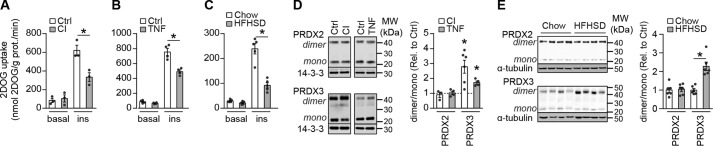
**Adipocyte insulin resistance is associated with increased mitochondrial oxidants.** Insulin resistance was induced in 3T3-L1 adipocytes by treatment with 10 nm insulin (*CI*) for 24 h (*A* and *D*) or 2 ng/ml TNFα (*TNF*) for 96 h (*B* and *D*) and in adipose tissue by 14 days of HFHSD feeding (*C* and *E*). *A–C*, 3T3-L1 adipocytes (*A* and *B*) and adipose tissue explants (*C*) were serum-starved for 2 h and assayed for 2DOG uptake following incubation without (*basal*) or with insulin (100 nm (*A* and *B*) or 10 nm (*C*)) for 20 min (*n* = 3 (*A*), *n* = 4 (*B*), and *n* = 5 (*C*), mean ± S.E. (*error bars*), two-sample *t* test; *, *p* < 0.05). *D* and *E*, PRDX2 and -3 dimer/monomer ratios were assessed by immunoblot. Immunoblots were quantified by densitometry and normalized to insulin-sensitive controls (indicated by the *dotted line* in *D*) (*n* = 3–6 (*D*) and *n* = 4 (*E*), mean ± S.E., two-sample *t* test; *, *p* < 0.05).

We have previously reported that multiple *in vitro* models of insulin resistance in cultured adipocytes and myotubes are associated with increased mitochondrial O_2_^˙̄^, as assessed by hydroethidine-derived fluorescence (4). However, these studies did not exclude the possibility that there were also increased oxidants in other cellular compartments, such as the cytosol. To assess whether insulin resistance is associated with increased oxidants specifically in mitochondria, we studied the cytosolic and mitochondrial hydroperoxide burden in two *in vitro* models and one *in vivo* model of adipocyte insulin resistance. Treatment of 3T3-L1 adipocytes with 10 nm insulin for 24 h to mimic hyperinsulinemia or with 2 ng/ml TNFα for 96 h to mimic chronic pro-inflammatory signaling impaired insulin-stimulated 2-deoxyglucose (2DOG) uptake (Fig. 1, *A* and *B*). Similarly, 14-day high-fat high-sucrose diet (HFHSD) feeding, which is sufficient to induce maximal adipose tissue insulin resistance (24, 25), impaired insulin-stimulated 2DOG uptake into adipose tissue explants (Fig. 1*C*). We assessed mitochondrial and cytosolic relative H_2_O_2_ exposure using the dimeric state of location-specific peroxiredoxins as a surrogate. 2-Cys PRDXs form disulfide-linked homodimers upon oxidation by hydroperoxides, such as H_2_O_2_, and are slowly reduced to their monomer by thioredoxin. Therefore, the PRDX dimer/monomer ratio acts as a surrogate for H_2_O_2_ content (26), although changes in thioredoxin activity and hydroperoxides other than H_2_O_2_ will also contribute. PRDX2 and PRDX3 are localized in the cytosol and mitochondria, respectively, so the dimer/monomer ratio of these isoforms is indicative of H_2_O_2_ content at these specific locations. The PRDX2 dimer/monomer ratio was unchanged in all insulin resistance models (Fig. 1, *D* and *E*), indicating that cytosolic peroxide levels were unchanged. In contrast, the PRDX3 dimer/monomer ratio was increased by 1.8–2.8-fold in *in vitro* models (Fig. 1*D*) and by 2.3-fold in *in vivo* models (Fig. 1*E*). These data suggest that insulin resistance is characterized by an oxidative burden specifically in mitochondria.

### Mitochondria-targeted paraquat elevates mitochondrial superoxide and hydrogen peroxide

**Figure 2.**
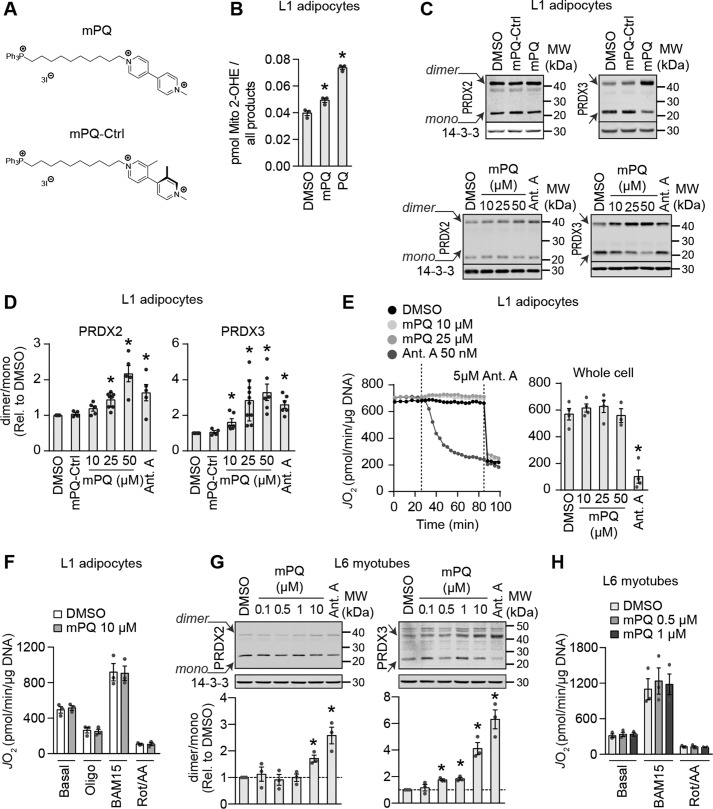
**Mitochondria-targeted paraquat increases mitochondrial superoxide and hydrogen peroxide without inhibiting cellular respiration.** *A*, chemical structures of MitoPQ (*mPQ*) and an inactive analogue MitoPQ-Ctrl (*mPQ-Ctrl*) used in this study. *B*, concentration of 2-hydroxyethidium derivative of MitoSox relative to all products of MitoSOX in cells treated with 25 μm MitoPQ or 5 mm paraquat (*PQ*) for 2 h as indicated (*n* = 4, mean ± S.E. (*error bars*), two-sample *t* test corrected for multiple comparisons; *, *p* < 0.05 *versus* DMSO-treated cells). *C* and *D*, PRDX2 and -3 dimer/monomer ratios in 3T3-L1 adipocytes treated with DMSO, 25 μm MitoPQ-Ctrl, or MitoPQ (*C*, *top*) or increasing doses of MitoPQ (*C*, *bottom*) were assessed by immunoblotting. Immunoblots were quantified by densitometry and normalized to DMSO-treated cells (*D*) (indicated by the *dotted line*) (PRDX2, *n* = 3–5; PRDX3, *n* = 3–6; mean ± S.E., one-sample *t* test; *, *p* < 0.05). *E*, oxygen consumption was measured in 3T3-L1 adipocytes cultured in glucose-containing medium. *Dotted lines* on the trace (*left*) indicate the time drugs were added. Stable rates of oxygen consumption (*J*_O2_) were compared between conditions (*n* = 4–5, mean ± S.E., two-sample *t* test corrected for multiple comparisons; *, *p* < 0.05). *F*, oxygen consumption was measured in 3T3-L1 adipocytes in galactose-containing medium. Following basal measurements, cells were sequentially treated with oligomycin (*Oligo*), BAM15 (*uncoupler*), and rotenone/antimycin A (*Rot/AA*) (*n* = 3, mean ± S.E., two-sample *t* test corrected for multiple comparisons). *G*, PRDX2 and -3 dimer/monomer ratios were assessed in L6 myotubes treated as indicated by immunoblotting. Immunoblots were quantified by densitometry and normalized to untreated cells (indicated by the *dotted line*) (PRDX2, *n* = 4; PRDX3, *n* = 4; mean ± S.E., one-sample *t* test; *, *p* < 0.05). *H*, oxygen consumption was measured in L6 myotubes in glucose-containing medium. Following basal measurements, cells were sequentially treated with, oligomycin, BAM15, and rotenone/antimycin A (*n* = 3, mean ± S.E., two-sample *t* test corrected for multiple comparisons).

Mitochondrial oxidants appear to play a causal role in insulin resistance because pharmacological (4) or genetic (3–5) scavenging of these oxidants has been shown to improve insulin action in adipose and muscle tissue. However, even in these examples, it is challenging to disentangle mitochondrial oxidative stress from mitochondrial dysfunction, as it is not clear how scavenging of these oxidants influences mitochondrial function. To test whether mitochondrial oxidants are sufficient to cause insulin resistance, we endeavored to establish a system where we could acutely increase mitochondrial oxidant production independently of other changes, such as mitochondrial dysfunction. To this end, here we describe the use of MitoPQ (Fig. 2*A*) (22).

MitoPQ has previously been reported to increase oxidants in isolated mitochondria and in mitochondria of C2C12 myoblasts (22). We tested whether incubation of 3T3-L1 adipocytes with MitoPQ increased mitochondrial O_2_^˙̄^ using LC-MS/MS to quantify the conversion of mito-hydroethidine to mito-2-hydroxyethidium (27), a superoxide-specific product. MitoPQ increased mito-2-hydroxyethidium concentrations compared with cells treated with a vehicle control (Fig. 2*B*), as did a high dose of paraquat, which was included as a positive control (Fig. 2*B*). We next tested whether increased mitochondrial O_2_^˙̄^ was due to the active paraquat moiety, and not a by-product of, for example, the triphenylphosphonium lipophilic cation, which mediates localization to the mitochondria. We tested this by using an inactive MitoPQ analogue (MitoPQ-control (MitoPQ-Ctrl); Fig. 2*A*), which is localized to mitochondria but does not generate O_2_^˙̄^ because the pyridine rings of the viologen component are not coplanar and thus cannot stabilize a radical cation effectively (28). Because O_2_^˙̄^ is rapidly dismutated to H_2_O_2_ in the presence of SOD2, we used PRDX dimerization as a proxy for subcellular O_2_^˙̄^ generation. Treatment of 3T3-L1 adipocytes with MitoPQ for 2 h increased the PRDX2 and PRDX3 dimer/monomer ratio (Fig. 2, *C* and *D*). Although MitoPQ enriches within the mitochondrial matrix and we expected to detect increased O_2_^˙̄^ and resulting H_2_O_2_ in the matrix (Fig. 2 (*B–D*), PRDX3), we also expected increased cytosolic H_2_O_2_ under conditions of high mitochondrial H_2_O_2_ due to diffusion of H_2_O_2_ from mitochondria (Fig. 2 (*C* and *D*), PRDX2). MitoPQ-Ctrl did not alter the dimerization status of either PRDX2 or -3 (Fig. 2, *C* and *D*), suggesting that MitoPQ-induced changes in the PRDX3 dimer/monomer ratio were due to redox cycling by the active paraquat moiety.

We next established the dose of MitoPQ that specifically induced PRDX3 dimerization without affecting cytosolic PRDX2 in 3T3-L1 adipocytes treated with MitoPQ for 2 h to mimic the subcellular oxidative burden in insulin resistance (Fig. 1). 10 μm MitoPQ increased PRDX3 dimer/monomer ratio in adipocytes without a change in PRDX2 (Fig. 2, *C* and *D*). As a control, we included antimycin A, which is an inhibitor of complex III that increases O_2_^˙̄^ production (29) and induces insulin resistance (4). Antimycin A increased PRDX3 dimerization in 3T3-L1 adipocytes (Fig. 2, *C* and *D*) but also increased PRDX2 dimerization, albeit to a lesser extent (Fig. 2, *C* and *D*). Thus, MitoPQ mimicked antimycin A by increasing mitochondrial O_2_^˙̄^ and H_2_O_2_ (hereafter referred to collectively as mitochondrial oxidants).

### Mitochondria-targeted paraquat does not alter whole-cell respiration

A potential major advantage of using MitoPQ over alternative small molecules, such as antimycin A, that increase mitochondrial oxidants by inhibiting the ETC is that it may be possible to disentangle the role of mitochondrial oxidants from mitochondrial dysfunction in insulin resistance. To assess whether MitoPQ could increase mitochondrial oxidants without affecting mitochondrial function, we monitored oxygen consumption rates in 3T3-L1 adipocytes. MitoPQ did not affect oxygen consumption in adipocytes at any dose tested, whereas 50 nm antimycin A inhibited mitochondrial respiration (Fig. 2*E*). To further test whether we could detect any impairment in mitochondrial respiration with MitoPQ, we pretreated adipocytes with MitoPQ for 90 min and assessed basal and maximal respiratory rates in galactose instead of glucose to force ATP production via mitochondria (30). Under these conditions, 10 μm MitoPQ still did not affect basal or uncoupled/maximal respiratory rates (Fig. 2*F*), indicating that we were not impairing mitochondrial respiration.

We next extended our oxidant and bioenergetic analyses to the L6 skeletal muscle cell line. L6 myotubes were more sensitive to MitoPQ than adipocytes (Fig. 2*G*), so that MitoPQ increased PRDX3 dimerization in L6 myotubes at ≥0.5 μm MitoPQ (Fig. 2*G*, *right*), and the PRDX2 dimer/monomer ratio was unaffected by MitoPQ at doses up to 10 μm (Fig. 2*G*, *left*). Antimycin A increased both PRDX2 and PRDX3 dimerization (Fig. 2*G*). As in adipocytes, MitoPQ did not impair basal or maximal respiration of myotubes (Fig. 2*H*). Together, these data show that MitoPQ specifically increased mitochondrial oxidants in 3T3-L1 adipocytes and L6 myotubes without increasing cytosolic oxidants or impairing respiratory function or capacity.

### Mitochondria-targeted paraquat inhibits insulin-stimulated GLUT4 translocation

**Figure 3.**
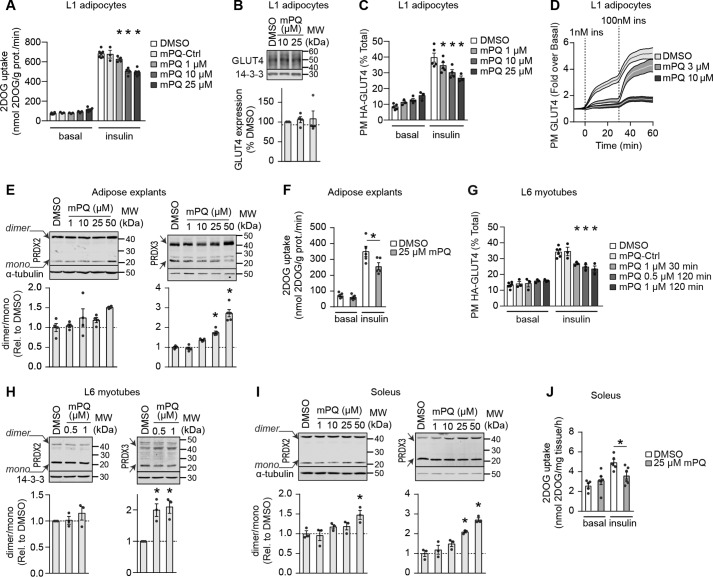
**Mitochondria-targeted paraquat induces insulin resistance.** *A*, 3T3-L1 adipocytes were assayed for 2DOG uptake following treatment with DMSO, MitoPQ-Ctrl, or MitoPQ at specified doses for 2 h and incubated without (basal) or with 100 nm insulin for 20 min (*n* = 3–4, mean ± S.E. (*error bars*), two-sample *t* test corrected for multiple comparisons; *, *p* < 0.05 *versus* insulin-stimulated DMSO control). *B*, GLUT4 levels were assessed by immunoblot. Immunoblots were quantified by densitometry and expressed relative to DMSO-treated cells (indicated by a *dotted line*) (*n* = 4, mean ± S.E., one-sample *t* test). *C*, 3T3-L1 adipocytes expressing HA-GLUT4 were treated as indicated and assessed for plasma membrane–localized HA-GLUT4 with and without insulin stimulation (*n* = 3–5, mean ± S.E., two-sample *t* test corrected for multiple comparisons; *, *p* < 0.05 *versus* insulin-stimulated DMSO control). *D*, 3T3-L1 adipocytes expressing pHlourin-GLUT4 were treated as indicated and assessed for plasma membrane–localized pHlourin-GLUT4 by live-cell total internal fluorescence microscopy as described under “Experimental procedures” (*n* ≥ 35 cells/condition from two biological replicates, mean ± S.E.). *E*, epididymal adipose explants were incubated with MitoPQ at the indicated doses for 2 h, and PRDX2 and -3 dimer/monomer ratios were assessed by immunoblotting. Immunoblots were quantified by densitometry and normalized to DMSO-treated explants (*n* = 3–4, mean ± S.E., two-sample *t* test corrected for multiple comparisons; *, *p* < 0.05 *versus* DMSO control). *F*, adipose tissue explants were incubated for 2 h in the presence of DMSO or 25 μm MitoPQ and assayed for 2DOG uptake following incubation without (basal) or with 10 nm insulin for 20 min (*n* = 5, mean ± S.E., two-sample *t* test; *, *p* < 0.05 *versus* insulin-stimulated DMSO control). *G*, L6 myotubes expressing HA-GLUT4 were treated with DMSO, MitoPQ-Ctrl, or MitoPQ at the specified doses, and insulin responses were assessed by determining plasma membrane–localized HA-GLUT4 (*n* = 3–5, mean ± S.E., two-sample *t* test corrected for multiple comparisons; *, *p* < 0.05 *versus* insulin-stimulated DMSO control). Incubation times with MitoPQ were as indicated. *H*, PRDX2 and -3 dimer/monomer ratios were assessed in L6 myotubes treated with DMSO or MitoPQ for 30 min by immunoblot. Immunoblots were quantified by densitometry and normalized to untreated cells (indicated by the *dotted line*) (*n* = 5, mean ± S.E., one-sample *t* test; *, *p* < 0.05). *I*, isolated soleus muscle was incubated with DMSO or MitoPQ at the indicated doses for 1 h, and PRDX2 and -3 dimer/monomer ratios were assessed by immunoblotting. Immunoblots were quantified by densitometry and normalized to DMSO-treated muscle (*n* = 3–4, mean ± S.E., two-sample *t* test corrected for multiple comparisons; *, *p* < 0.05 *versus* DMSO control). *J*, isolated soleus muscle was incubated for 1 h in the presence of DMSO or 25 μm MitoPQ and assayed for 2DOG uptake following incubation without (basal) or with 10 nm insulin for 20 min (*n* = 5–6, mean ± S.E., two-sample *t* test; *, *p* < 0.05 *versus* insulin-stimulated DMSO control).

We next tested whether MitoPQ could induce insulin resistance in cultured adipocytes and myotubes. Because 10 μm MitoPQ recapitulated the mitochondria-specific increase in oxidants in insulin resistance models and this dose of MitoPQ did not interfere with whole-cell respiration (Fig. 2, *E* and *F*) in adipocytes, we used this dose of MitoPQ to assess the effect of increased mitochondrial oxidants on insulin-regulated glucose transport. Treatment of adipocytes for 2 h with 10 μm MitoPQ inhibited insulin-stimulated 2DOG uptake by 26%, and a higher dose of MitoPQ (25 μm) had no additional effect (Fig. 3*A*). In contrast, MitoPQ-Ctrl did not affect insulin-stimulated 2DOG uptake (Fig. 3*A*), suggesting that impaired insulin action was a consequence of MitoPQ-induced mitochondrial oxidants. We next tested various aspects of insulin-stimulated glucose transport to identify the specific process affected by MitoPQ. Insulin stimulates glucose uptake via the glucose transporter GLUT4. Expression of GLUT4 in adipocytes was unchanged by MitoPQ treatment (Fig. 3*B*), suggesting that MitoPQ might impair insulin-stimulated GLUT4 translocation to the plasma membrane. To test this, we assayed cell-surface GLUT4 content using two complementary GLUT4 reporter constructs: hemagglutinin (HA)-GLUT4 and pHluorin-GLUT4 in fixed-cell and live-cell assays, respectively. These constructs were modified within the primary exofacial loop of GLUT4 to permit assessment of GLUT4 plasma membrane content through the accessibility of the HA epitope in nonpermeabilized cells (Fig. 3*C*) or due to the increase in fluorescence of pHluorin when exposed to pH 7.4 of the extracellular buffer in live-cell assays (Fig. 3*D*). In the fixed-cell assay, insulin augmented HA-GLUT4 PM levels by 4.5-fold, and this was inhibited by 26% in the presence of 10 μm MitoPQ (Fig. 3*C*). Live imaging of pHluorin-GLUT4 trafficking revealed that both 3 and 10 μm MitoPQ impaired insulin-stimulated GLUT4 exocytosis to the plasma membrane (Fig. 3*D*). We next tested the effect of MitoPQ on insulin responses in primary adipose explants. As in cultured cells (Fig. 2), MitoPQ (25 and 50 μm) increased the PRDX3 dimer/monomer ratio without altering that of PRDX2 (Fig. 3*E*), indicative of oxidant production in mitochondria. Incubation with 25 μm MitoPQ impaired insulin-stimulated 2DOG uptake in adipose explants (Fig. 3*F*).

As in adipocytes and adipose tissue, MitoPQ induced insulin resistance in L6 myotubes as measured by HA-GLUT4 translocation (Fig. 3*G*) at doses that selectively increased oxidants in the mitochondria but not in the cytosol and that had no detectable effect on mitochondrial respiration (Fig. 2, *G* and *H*). The degree of insulin resistance was similar at 0.5 and 1 μm MitoPQ and at higher doses (data not shown). This was specific to MitoPQ with an active paraquat moiety because the MitoPQ-Ctrl had no effect (Fig. 3*G*). We also tested whether MitoPQ could impair GLUT4 translocation more acutely in L6 myotubes to test whether more acute induction of mitochondrial oxidants could cause insulin resistance. Incubation with 1 μm MitoPQ for 30 min increased the PRDX3 dimer/monomer ratio to a similar extent as measured after a 2-h incubation (Figs. 2*G* and 3*H*), and this was accompanied by impaired insulin-stimulated HA-GLUT4 translocation (Fig. 3*G*). We extended our work in muscle to isolated soleus to determine whether specific induction of mitochondrial oxidants with MitoPQ also caused insulin resistance in primary muscle preparations (Fig. 3, *I* and *J*). Here, using a dose of MitoPQ that specifically increased PRDX3 dimerization (25 μm), we also observed impaired insulin-stimulated 2DOG uptake (Fig. 3*J*).

Together, these data show that MitoPQ acutely impairs insulin-stimulated glucose uptake by blocking GLUT4 trafficking, supporting a role for mitochondrial oxidants, independent of mitochondrial function, in the induction of insulin resistance in adipose and muscle tissue.

### Mitochondria-targeted paraquat induces insulin resistance without defects in canonical insulin signaling

**Figure 4.**
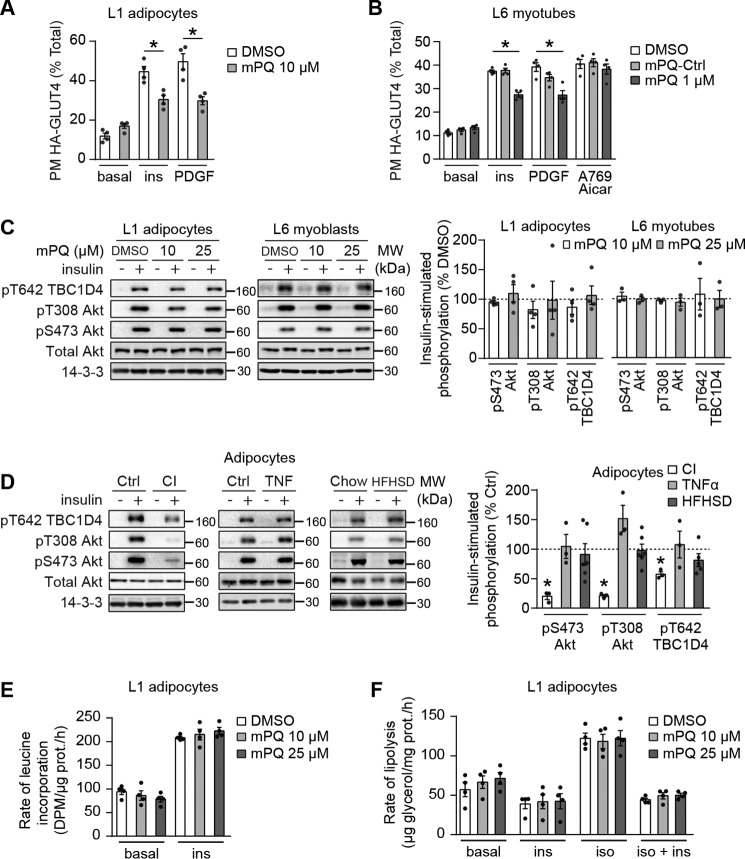
**Mitochondria-targeted paraquat induces insulin resistance downstream of Akt.** *A* and *B*, 3T3-L1 adipocytes (*A*) or L6 myotubes (*B*) overexpressing HA-GLUT4 and PDGFR were serum-starved, treated with DMSO or the indicated doses of MitoPQ (*mPQ*) for 2 h, and assessed for plasma membrane–localized HA-GLUT4 following incubation without (*basal*) or with 100 nm insulin, 20 ng/ml PDGF, or 2 mm AICAR and 100 μm A-769662 (*A769*) for 20 min where indicated (*n* = 4, mean ± S.E. (*error bars*), two-sample *t* test corrected for multiple comparisons; *, *p* < 0.05; comparisons as indicated). *C*, 3T3-L1 adipocytes (*left*) and L6 myotubes (*right*) were serum-starved and treated with DMSO or the indicated doses of MitoPQ for 2 h and insulin where indicated. Phosphorylation status of indicated sites was assessed by immunoblot. Immunoblots were quantified by densitometry and normalized to insulin-treated control cells (indicated by the *dotted line*) (*n* = 4, mean ± S.E., one-sample *t* test; *, *p* < 0.05). *D*, insulin resistance was induced in 3T3-L1 adipocytes by treatment with 10 nm insulin (*CI*) for 24 h or 2 ng/ml TNFα (*TNF*) for 96 h and in adipose tissue by 14-day HFHSD feeding. 3T3-L1 adipocytes or adipose tissue explants were serum-starved for 2 h and incubated without or with 100 nm insulin for 20 min. Phosphorylation status of the indicated sites was assessed by immunoblotting. Immunoblots were quantified by densitometry and normalized to insulin-treated control cells (indicated by the *dotted line*) (insulin and TNFα, *n* = 3; HFHSD, *n* = 6; mean ± S.E., one-sample *t* test; *, *p* < 0.05). *E* and *F*, 3T3-L1 adipocytes were serum-starved and treated with DMSO or the indicated doses of MitoPQ for 2 h, and protein synthesis was assessed by incorporation of [^3^H]leucine into protein in the presence and absence of 100 nm insulin (*n* = 4, mean ± S.E., two-sample *t* test corrected for multiple comparisons) (*E*), or lipolysis was assessed via release of glycerol into culture medium after stimulation with 1 nm isoproterenol with and without incubation with 100 nm insulin (*n* = 4, mean ± S.E., two-sample *t* test corrected for multiple comparisons) (*F*).

Impaired insulin signaling through IRS1 to Akt is often observed in insulin resistance (31, 32) and has been hypothesized to be upstream of defects in GLUT4 translocation. Indeed, it has been proposed that mitochondrial oxidants cause insulin resistance through the activation of stress kinases, such as c-Jun N-terminal kinases (JNKs), and subsequent phosphorylation and down-regulation of IRS1 (33, 34). We next directly tested the requirement for IRS1 in MitoPQ-induced insulin resistance in adipocytes and L6 myotubes. To achieve this, we utilized 3T3-L1 adipocytes ectopically expressing platelet-derived growth factor receptor (PDGFR). This is a useful system because PDGF stimulates Akt phosphorylation and GLUT4 translocation in a manner similar to insulin but independently of IRS1 (35). MitoPQ inhibited both insulin- and PDGF-stimulated GLUT4 translocation (Fig. 4*A*), indicating that IRS1 is not the target of the mitochondrial oxidant-induced defect in GLUT4 translocation. The same phenomenon was observed in L6 myotubes (Fig. 4*B*).

These data implicated events downstream of IRS1 in MitoPQ-induced insulin resistance. We next tested whether insulin signaling to Akt or the Akt substrate most associated with GLUT4 translocation, TBC1D4, was defective in MitoPQ-treated cells. In both 3T3-L1 adipocytes and L6 myotubes, insulin treatment increased phosphorylation of Akt at activating sites and of TBC1D4 at Thr-642 (Fig. 4*C*). Insulin signaling to these sites was not affected by 10 or 25 μm MitoPQ in either cell line (Fig. 4*C*). Therefore, impaired insulin-stimulated 2DOG uptake or GLUT4 translocation in response to MitoPQ in 3T3-L1 adipocytes (Fig. 3, *A* and *C*), adipose explants (Fig. 3*F*), and L6 myotubes (Fig. 3*G*) occurred without any detectable defect in the major signaling proteins thought to regulate GLUT4 trafficking in response to insulin. These data, in combination with the lack of a consistent signaling defect in *in vivo* and *in vitro* models of adipocyte insulin resistance (Fig. 4*D*), support previous reports indicating that it is unlikely that insulin resistance is due to a defect in proximal components of the insulin signaling pathway (24, 35–37).

### Mitochondria-targeted paraquat selectively inhibits insulin-stimulated glucose transport

We next determined whether the effects of MitoPQ on GLUT4 translocation were specific to insulin. Previously, it has been shown that exercise- or AMPK-induced GLUT4 translocation in muscle is not impaired in insulin resistance (38–40). Thus, we assessed the effects of MitoPQ on AMPK-stimulated GLUT4 translocation in L6 myotubes. Treatment of L6 myotubes with the AMPK agonists aminoimidazole-4-carboxamide ribonucleotide (AICAR) and A-769662 increased cell-surface HA-GLUT4 to a similar extent as observed with insulin (Fig. 4*B*). However, in contrast to that seen with insulin, MitoPQ had no significant effect on AMPK-induced GLUT4 translocation in L6 myotubes. Therefore, the defects in insulin-stimulated GLUT4 translocation in MitoPQ-treated cells and in insulin resistance models appear to be specific to insulin action. Furthermore, the absence of any defect in AMPK-stimulated GLUT4 trafficking provides a further similarity between MitoPQ-induced insulin resistance and most other models described previously (38–40).

We next investigated the effects of MitoPQ on other insulin-regulated processes, as insulin resistance has been reported to be somewhat selective (23, 41). In contrast to its effects on glucose uptake and GLUT4 trafficking, MitoPQ had no significant effect on insulin-stimulated protein synthesis or insulin-dependent regulation of lipolysis in adipocytes at 100 nm insulin (Fig. 4, *E* and *F*). Therefore, consistent with other physiological models of insulin resistance, such as the diet-induced obese mouse, insulin-stimulated GLUT4 translocation was sensitive to MitoPQ under conditions where other insulin/Akt-regulated processes were unaffected.

## Discussion

**Figure 5.**
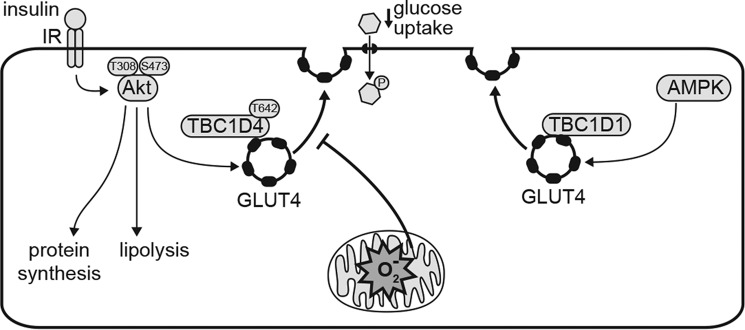
**Mitochondrial oxidants induces selective insulin resistance.** MitoPQ generates oxidants (O_2_^˙̄^ and H_2_O_2_; denoted by *O*_2_^˙̄^) in the mitochondrial matrix without impairing oxidative phosphorylation. Mitochondrial oxidants inhibit insulin-stimulated GLUT4 translocation and glucose uptake in adipocytes and myocytes, but AMPK-stimulated GLUT4 translocation is not impaired. MitoPQ did not impair insulin signaling to TBC1D4 or Akt-mediated activation of protein synthesis and inhibition of lipolysis, indicating that mitochondrial oxidants impair processes downstream of TBC1D4 phosphorylation in the insulin-regulated GLUT4 trafficking pathway.

It has been reported previously that mitochondrial oxidants cause insulin resistance, but a method to specifically induce oxidants at this site without affecting mitochondrial respiration has been lacking. In this study, we have obtained three novel insights into the role of superoxide and oxidative stress in insulin resistance. First, using assessment of the oligomeric state of compartment-specific PRDXs, we provide compelling evidence that insulin resistance is associated with a specific increase in mitochondrial hydroperoxides. Second, we show that MitoPQ, which selectively induces mitochondrial oxidants (O_2_^˙̄^/H_2_O_2_) without inhibiting mitochondria respiration, rapidly induces insulin resistance. Third, we observed insulin resistance in response to MitoPQ, as measured by defective insulin-stimulated 2DOG uptake and GLUT4 translocation in cultured cells and in tissue without any demonstrable defect in proximal components of the insulin signaling pathway. Insulin resistance caused by MitoPQ resembled the mode of insulin resistance seen in many other models, including its relative specificity for glucose uptake over other insulin-regulated processes and its selectivity for insulin-regulated glucose uptake, as it had no significant impact on the ability of other agonists, such as AICAR. These findings provide support for the induction of an acute retrograde pathway from mitochondrial oxidants that converges upon the insulin-responsive GLUT4 machinery in cytosol in a highly selective manner (Fig. 5).

One challenge in studying insulin resistance is that most insults that lead to insulin resistance have a multitude of effects, making it difficult to delineate causal factors. For example, cellular stresses associated with insulin resistance, such as increased ceramides and endoplasmic reticulum stress, which have been reported to independently cause insulin resistance, also induce mitochondrial oxidative stress (11, 42). The interconnectivity of these stresses and wide-ranging transcriptional changes often associated with insults that induce insulin resistance (43) makes it difficult to pinpoint the major causes of insulin resistance in each case. There is accumulating evidence that oxidants are elevated in insulin resistance (44) and that these oxidants originate from mitochondria (2–5). Using PRDX dimerization as a readout, we report that increased mitochondrial H_2_O_2_, in the absence of cytosolic H_2_O_2_, is a common feature of three distinct models of adipocyte insulin resistance. It should be noted that PRDXs can react with other hydroperoxides, such as lipid and protein hydroperoxides, so we also cannot exclude a role for these oxidants in insulin resistance. Here we report that acute increases in mitochondrial oxidants via MitoPQ are sufficient to drive insulin resistance in cultured adipocytes and myotubes and in adipose tissue and soleus muscle *ex vivo*. This supports data from transgenic mouse models, which show that enhanced scavenging of mitochondrial O_2_^˙̄^ (via SOD2) or H_2_O_2_ (via mitochondria-targeted catalase or PRDX3) improved insulin action (3–5) or glucose tolerance (45). Together, these data suggest that mitochondrial oxidants are necessary and sufficient to induce insulin resistance in adipocyte and muscle cells, although there remains conflicting evidence as to the relative contribution and roles of O_2_^˙̄^ and H_2_O_2_ (4, 46).

We have been interested in mitochondrial oxidants and their role in insulin resistance for some time, yet many perturbations used previously have a range of potential off-target effects. Small molecules that increase O_2_^˙̄^ production in mitochondria often block the electron transport chain, which could have many off-target effects. For example, we have previously used antimycin A to induce O_2_^˙̄^ and insulin resistance in an oxidant-dependent manner (4), but this compound acts by impairing mitochondrial respiration. Genetic models, including knockout of oxidant scavenging systems in the mitochondria such, as SOD2 or PRDX3, or expression of mitochondria-targeted catalase, tend to be longer term and may be associated with adaptive responses (*e.g.* mitochondrial function) that can modulate insulin sensitivity (20, 21). Thus, there has been an enormous demand for more precise strategies to perturb mitochondrial oxidant homeostasis without generating off-target effects, such as inhibiting mitochondrial function. The generation of MitoPQ was a major breakthrough because it offers a means to acutely increase mitochondria O_2_^˙̄^ content without directly impairing mitochondrial respiration. In the current study, we comprehensively analyzed the effect of MitoPQ on insulin action in cultured adipocyte and muscle cells and show that this agent provides an ideal model system for studying insulin resistance because it selectively generated O_2_^˙̄^/H_2_O_2_ in mitochondria and not the cytosol without impairing whole-cell respiration in either 3T3-L1 adipocytes or L6 myotubes. Further, MitoPQ induced insulin resistance in primary adipose and muscle tissue and recapitulated many of the features of insulin resistance, including impairment of insulin-regulated glucose transport independently of defects in insulin signaling without inhibiting AMPK-induced GLUT4 translocation. Hence, this provides compelling support to implicate mitochondrial oxidants as a major cause of insulin resistance, and this agent will provide an invaluable tool for assessing how mitochondrial oxidants affect insulin action *in vivo* and in other tissues, such as liver, and for studying the mechanism by which mitochondrial oxidant stress induces insulin resistance.

Despite the importance of mitochondrial oxidants in insulin resistance, there are a number of key unanswered questions. For example, what is the source of increased oxidants in mitochondria in insulin resistance? Although there is some evidence that fatty acid oxidation may contribute (5), a number of sites of O_2_^˙̄^ production in mitochondria have been described (47), and O_2_^˙̄^ can be produced in the intramembrane space or the matrix. However, because MitoPQ, which produces matrix O_2_^˙̄^, and antimycin A, which produces O_2_^˙̄^ in both the intermembrane space and matrix (48), both cause insulin resistance, our data suggest that matrix O_2_^˙̄^ signals to induce insulin resistance. However, we cannot rule out a role for O_2_^˙̄^ produced in the intermembrane space. Indeed, it has recently been reported that the site of oxidant production has profound effects on the biological outcome (49), so it will be important to establish how oxidants are being produced in insulin resistance to better inform the development of the most appropriate targeted therapies.

Another outstanding question is how mitochondrial oxidants signal to cause insulin resistance. The rapid onset of insulin resistance upon treatment with MitoPQ argues for an acute signaling mechanism between mitochondria and insulin signaling/GLUT4 trafficking and that mitochondrial oxidants are a distal component in the ontogeny of insulin resistance. One possibility is that mitochondrial oxidants directly impair insulin action in the cytosol through oxidation of specific proteins. However, we have no evidence for altered cytosolic H_2_O_2_ at the doses of MitoPQ used to induce insulin resistance or in models of insulin resistance in adipocytes, although this does not rule out a role for a localized oxidizing environment around mitochondria. Alternatively, a mitochondria-derived H_2_O_2_-responsive signal cascade was recently reported (50), and this would fit with previous reports that oxidative stress activation of JNKs may play a role in insulin resistance (33). However, the proposed mechanism for JNK-mediated insulin resistance via Ser/Thr hyperphosphorylation of IRS1 (34) is inconsistent with our data showing that MitoPQ-induced insulin resistance is IRS1-independent. Reduced Akt and TBC1D4 phosphorylation has been observed in some models of insulin resistance (6, 31, 32, 35). However, our studies with MitoPQ, where we observed insulin resistance without defects in Akt/TBC1D4, raise questions over whether such changes in insulin signaling are the primary driver of insulin resistance. Indeed, we and others have reported normal Akt activation in insulin-resistant cells and tissues (24, 35, 51). Furthermore, insulin-stimulated phosphorylation of Akt is defective in muscle from insulin resistant humans, but this defect does not translate into defective phosphorylation of a range of Akt substrates (52). We propose that if mitochondrial oxidants impair insulin signaling to GLUT4 (*e.g.* via JNK or other oxidant-activated kinases), they probably do so by targeting processes more specific to insulin regulation of GLUT4 trafficking (Fig. 4*G*), and this requires further study. Finally, because GLUT4 trafficking is highly dynamic, transiting between specialized GLUT4 storage vesicles and the *trans*-Golgi network, it may be that mitochondrial oxidants impair GLUT4 localization to GLUT4 storage vesicles. However, because AMPK-mediated GLUT4 translocation in L6 myotubes was unaffected by MitoPQ, this model would depend on insulin and AMPK targeting a distinct pool of GLUT4 (53), with the insulin-responsive pool being a specific target of mitochondrial signaling. Mitochondria are reported to make contact with numerous organelles, including endosomes, so mitochondria may directly communicate with GLUT4 trafficking machinery (*e.g.* through protein oxidation) via these interactions.

In summary, we have found that the oxidative stress burden in insulin resistance is specific to mitochondria and that selectively inducing mitochondrial oxidants independently of changes in oxidative phosphorylation is sufficient to induce insulin resistance in adipocytes and muscle cells.

## Experimental procedures

### Animals

Eight-week-old male C57BL/6J mice were purchased from the animal resources center (Perth, Australia). The animals were kept in a temperature-controlled environment (22 ± 1 °C) on a 12-h light/dark cycle with free access to food and water. Mice were fed *ad libitum* for a period of 14 days with a standard laboratory diet (chow) (13% calories from fat, 22% calories from protein, and 65% calories from carbohydrate, 3.1 kcal/g; Gordon's Specialty Stock Feeds, Yanderra, Australia) or with a high-fat high-sucrose diet (47% of calories from fat, 21% calories from protein, and 32% calories from carbohydrates, 4.7 kcal/g). All experiments were carried out with the approval of the University of Sydney Animal Ethics Committee, following guidelines issued by the National Health and Medical Research Council of Australia.

### Cell culture

3T3-L1 fibroblasts obtained from the Howard Green laboratory (Harvard Medical School) were cultured and differentiated into adipocytes as described previously (54). 3T3-L1 adipocytes were used 8–10 days after initiation of differentiation. L6 myoblasts were cultured and differentiated into myotubes as described previously (53) and used for experiments 4–6 days after initiation of differentiation. For overexpression of HA-tagged GLUT4, fibroblasts or myoblasts were infected with pBABE-derived or pWZL-derived HA-GLUT4 retrovirus, and infected cells were selected for with puromycin (2 μg/ml) or G-418 (0.8 mg/ml) as appropriate. For overexpression of PDGFR, fibroblasts were infected with pBABE-derived PDGFR retrovirus, and infected cells were selected for with puromycin. For chronic insulin treatments, adipocytes were incubated with 10 nm insulin for 1 day, with medium replenished every 12 h. For induction of insulin resistance by TNFα, adipocytes were incubated with 2 ng/ml TNFα for 96 h with medium replenished every 24 h. For acute insulin treatments, 3T3-L1 adipocytes were serum-starved in DMEM supplemented with 0.2% BSA and Glutamax (basal-DMEM) with the indicated doses of MitoPQ (or vehicle control, DMSO) or antimycin A (or vehicle control, EtOH), for 30 min or 2 h, as indicated, followed by the addition of 100 nm insulin.

### Assays in adipose tissue explants

Epididymal fat depots were excised from mice. Fat pads were immediately transferred to warm DMEM, 2% BSA, 20 mm HEPES, pH 7.4, and minced into fine pieces. Explants were washed twice and incubated in DMEM, 2% BSA, 20 mm HEPES, pH 7.4, for 2 h.

For 2DOG uptake, explants were rinsed and incubated in Krebs–Ringer phosphate (KRP), 2% BSA. Insulin (10 nm) was added for 20 min, and glucose transport was initiated by the addition of ^3^H-labeled 2-deoxyglucose (0.25 μCi/sample, 50 μm) ([^3^H]2DOG) and [^14^C]mannitol (0.036 μCi/sample) for the final 5 min of the assay. Uptake was terminated with three rapid washes in ice-cold PBS, after which the cells were solubilized in radioimmune precipitation assay (RIPA) buffer (50 mm Tris-HCl, pH 7.5, 150 mm NaCl, 1% Triton X-100, 0.5% sodium deoxycholate, 0.1% SDS, 1 mm EDTA, and 10% glycerol) supplemented with protease inhibitors (Roche Applied Science). Samples were assessed for radioactivity by liquid scintillation spectroscopy, and glucose uptake was calculated after correcting the [^3^H]2DOG counts for extracellular [^14^C]mannitol counts and normalized for protein content following a BCA assay.

For experiments using MitoPQ, 25 μm MitoPQ or equivalent volume of vehicle (DMSO) was included during the 2-h incubation period in DMEM, 2% BSA and maintained throughout subsequent washes and incubation. Also, the 2DOG uptake assay was carried out in DMEM without glucose, 2% BSA rather than KRP, 2% BSA. Samples were processed as described above following termination of 2DOG uptake with three rapid washes in ice-cold PBS. For Western blot analysis, fat explants were treated with insulin after incubation in DMEM, 2% BSA, 20 mm HEPES, pH 7.4, for 2 h. Signaling was terminated with three rapid washes in ice-cold PBS, and cells were solubilized in RIPA buffer supplemented with protease inhibitors (Roche Applied Science).

### 2DOG uptake assay in soleus muscle

[^3^H]2DOG uptake into isolated soleus muscles was performed as described previously (55). Soleus muscles were excised, mounted, and preincubated for 1 h at 30 °C in Krebs Henseleit buffer containing 5.5 mm glucose, 2 mm pyruvate, and 0.1% BSA (KRH) containing DMSO (vehicle control) or 25 μm MitoPQ for 1 h. KRH was pregassed with carbogen (95% O_2_, 5% CO_2_). Glucose uptake was assessed following the addition of [^3^H]2DOG (final concentration 0.375 μCi/ml) and [^14^C]mannitol (0.05 μCi/ml) with or without 10 nm insulin or 2 mm AICAR (Toronto Research Chemicals). After 20 min, muscles were washed in ice-cold PBS and snap-frozen. Frozen muscles were incubated in 1 m KOH at 70 °C for 20 min. Tracer content was quantified by liquid scintillation spectroscopy, and glucose uptake was calculated after correcting the [^3^H]2DOG counts for extracellular [^14^C]mannitol counts and tissue weight.

### Assessment of peroxiredoxin dimerization

Cells, explants, or isolated soleus muscles were washed three times with ice-cold PBS that was pretreated with 10 μg/ml catalase for 1 h. Cells were incubated with 100 mm *N*-ethylmaleimide (NEM) in PBS for 10 min on ice to alkylate free cysteines. Cells and soleus muscles were lysed by sonication in 2% SDS in PBS-containing protease inhibitors (Roche Applied Science) and 100 mm NEM. Epididymal adipose explants were lysed by sonication in in 2× RIPA buffer containing protease inhibitors and 100 mm NEM. Samples were separated by nonreducing SDS-PAGE.

### Synthesis of MitoPQ-Ctrl and MitoPQ

The structures of MitoPQ and of the MitoPQ-Ctrl compound are shown in Fig. 2*A*. MitoPQ was synthesized as described (22). The synthesis and characterization of the MitoPQ control compound will be described in a future publication.

### Assessment of conversion of mito-hydroethidine (MitoSOX) to mito-2-hydroxyethidium

Conversion of MitoSOX to mito-2-hydroxyethidium was detected by LC/tandem MS (LC-MS/MS) as described previously (27) with modifications. 3T3-L1 adipocytes were incubated with 5 mm paraquat or 25 μm MitoPQ in DMEM/GlutMAX without FCS for a total of 2 h, and MitoSOX (Thermo Scientific) was added to 5 μm for the final 60 min. Cells were scraped in ice-cold ethanol and centrifuged at 17,000 × *g* for 15 min, 4 °C. 5 μl of supernatant was injected onto an Agilent 1290 UHPLC system connected to an Agilent 6490 triple-quadrupole mass spectrometer. Analytes were separated on a 2.6-μm Kinetex XB-C18 100 Å column (50 × 2.10 mm; Phenomenex) by gradient elution using mobile phase A (0.1% formic acid in water) and mobile phase B (0.1% formic acid in acetonitrile) at 0.4 ml/min. The gradient consisted of 25% mobile phase B from 0 to 2 min, 25–40% B from 2 to 7 min, 40–90% B from 7 to 8 min, 90% B from 8 to 11 min, and back to 25% B from 11 to 12 min. Flow was then directed into the triple quadrupole mass spectrometer with parameters set as follows: gas temperature = 290 °C; gas flow = 14 liters/min; nebulizer pressure = 35 p.s.i.; sheath gas heater = 350 °C; sheath gas flow = 11 liters/min; capillary voltage = 3,500 V. Detection of MitoSOX, mito-ethidium (Mito-E^+^), and mito-hydroxyethidium (Mito-2OHE^+^) was by multiple-reaction monitoring in positive ion mode using the above general MS parameters with fragmentor voltage at 380 V and cell accelerator voltage at 5 V. In each case, the fragment ions generated by collision-induced dissociation of the [M + H]^+^ or the [M + 2H]^+^ ions were used for quantification. Multiple-reaction monitoring settings for the target analytes were as follows (parent ion → fragment ion): MitoSOX (*m*/*z* 632.2 → 289) with collision energy (CE) = 25 V; Mito-E^+^ (*m*/*z* 315.7 → 289) with CE = 25 V; and Mito-2OHE^+^ (*m*/*z* 323.7 → 289) with CE = 25 V. MitoSOX was quantified against authentic commercial standard obtained from Invitrogen, whereas Mito-E^+^ and Mito-2OHE^+^ were quantified against standards synthesized from MitoSOX as described previously (27).

### Immunoblotting

Cells were washed three times with ice-cold PBS and homogenized in 2% SDS by sonication. Insoluble material was removed by centrifugation at 21,000 × *g*. Protein concentration was determined by bicinchoninic acid method (Thermo Scientific). 10 μg of protein was resolved by SDS-PAGE and transferred to PVDF membranes. Membranes were blocked in 5% skim milk powder in Tris-buffered saline for 1 h, followed by an overnight incubation at 4 °C with specific primary antibody solutions. Antibodies specific for pSer-473 Akt, pThr-308 AKT, pThr-642 TBC1D4, and Akt were from Cell Signaling Technology; GLUT4 antibody was made in-house; α-tubulin antibody was from Sigma; and 14-3-3β antibody was from Santa Cruz Biotechnology, Inc. Antibodies specific for PRDX2 were from Abcam, and PRDX3 antibody was from Abfrontier. Membranes were incubated with an appropriate secondary antibody for 1 h before signals were detected using ECL (Thermo Scientific or Millipore) on the Chemidoc MP (Bio-Rad). In some cases, IRDye700- or IRDye800-conjugated secondary antibodies were used and then scanned at the 700- and 800-nm channel using the Odyssey IR imager. Densitometry analysis of immunoblots was performed using ImageJ version 1.47.

### 2DOG uptake assay in 3T3-L1 adipocytes

2DOG uptake was determined as described previously (56). 3T3-L1 adipocytes in 24-well plates were serum-starved in basal-DMEM for 2 h with MitoPQ where indicated. Cells were then washed three times in warm PBS and incubated in KRP (0.6 mm Na_2_HPO_4_, 0.4 mm NaH_2_PO_4_, 120 mm NaCl, 6 mm KCl, 1 mm CaCl_2_, 1.2 mm MgSO_4_, and 12.5 mm Hepes (pH 7.4)) with 0.2% BSA. [^3^H]2DOG (PerkinElmer Life Sciences) (0.125 μCi/well) and 50 μm unlabeled 2DOG were added for 5 min. 2DOG uptake was terminated with three rapid washes in ice-cold PBS, after which cells were solubilized in 1% (w/v) Triton X-100 in PBS on a shaker for 1 h and assessed for radioactivity by scintillation counting using a β-scintillation counter. To determine nonspecific glucose uptake, cells were treated with 25 μm cytochalasin B 30 min before the addition of 2DOG. To measure insulin-sensitive glucose uptake, cells were stimulated with 10 nm insulin for 20 min, and 2DOG was added in the final 5 min of insulin stimulation. All data were normalized to protein content. Each condition was performed in triplicate.

### HA-GLUT4 assay

HA-GLUT4 levels at the plasma membrane were determined as described previously (54, 57). Briefly, cells were serum-starved for 2 h in basal-DMEM and stimulated with 100 nm insulin or 20 ng/ml PDGF for 20 min as indicated. To determine the amount of HA-GLUT4 at the plasma membrane, cells were fixed but not permeabilized and incubated with anti-HA antibody (Covance), followed by 20 μg/ml goat anti-mouse Alexa Fluor 488–conjugated secondary antibody (Life Technologies, Inc.). To determine the total amount of HA-GLUT4, cells in separate wells were fixed and permeabilized with 0.1% (w/v) saponin and stained for HA as above. Fluorescence (excitation 485 nm/emission 520 nm) was measured using a fluorescent microtiter plate reader (FLUOstar Galaxy, BMG LABTECH).

### Live-cell microscopy

Live-cell imaging was performed as described previously (58). Briefly, at 7 days of post-differentiation, adipocytes were trypsinized with 5× trypsin/EDTA for 5–10 min at 37 °C, washed twice with PBS, and resuspended in electroporation solution (20 mm Hepes, 135 mm KCl, 2 mm MgCl_2_, 0.5% Ficoll 400, 1% DMSO, 2 mm ATP, and 5 mm GSH, pH 7.6) with 5–20 μg of plasmid DNA (pHluorin-GLUT4). Cells were electroporated at 200 mV for 20 ms using an ECM 830 Square Wave Electroporation System (BTX Molecular Delivery Systems) and seeded into Matrigel-coated 35-mm glass-bottomed dishes (Ibidi). Adipocytes were maintained in DMEM supplemented with 10% FCS and 1% GlutaMAX until required. Healthy, transfected cells were identified by brightfield and fluorescence microscopy. 15–25 cells were imaged per experiment per condition. Cells were imaged every 60 s for 10 min before the addition of MitoPQ and imaged for a further 1.5 h. Insulin was added to 1 and 100 nm at the indicated times. Cells were analyzed using custom analysis pipelines written in Fiji (59).

### Mitochondrial bioenergetics

On day 7 of differentiation, 3T3-L1 adipocytes were trypsinized and seeded into Matrigel-coated XFp cell culture plates. L6 cells were differentiated into myotubes in Matrigel-coated XFp cell culture plates. For analysis of whole-cell respiration (60), cells were washed with warm PBS and incubated in phenol red–free DMEM containing 30 mm HEPES, pH 7.4, 10 mm NaHCO_3_, 1 mm glutamine, and 1 mm GlutaMAX containing 25 mm glucose or 25 mm galactose as indicated. The following injections were used for Fig. 2*D*: port A, MitoPQ to required dose, antimycin A to 50 nm, or vehicle control; port B-C, maintenance treatment from port A; port D, 10 μm antimycin A. For experiments in Fig. 2 (*E* and *H*), adipocytes or myotubes were preincubated with the specific dose of MitoPQ for 90 min. The following injections were used: port A, maintenance of MitoPQ or vehicle control; port B, 10 μg/ml oligomycin; port C, 10 μm BAM15 (61); port D, 5 μm rotenone and 10 μm antimycin A. Each cycle included 3 min of mixing time, 0 min of waiting time, and 2 min of measuring time, repeated until a steady *J*_O2_ reading was obtained after each injection. Nonmitochondrial respiration was taken as *J*_O2_ following 5 μm antimycin A. All resulting rates were normalized to DNA content, measured using Hoechst 33248 with a previously described Hoechst-staining protocol (60).

### Lipolysis assay

3T3-L1 adipocytes in 24-well plates were serum-starved in basal-DMEM for 2 h with MitoPQ where indicated. Cells were washed three times in warm PBS and incubated in KRP supplemented with 3.5% free fatty acid BSA (Sigma-Aldrich) and 5 mm glucose for 2 h. Cells were treated with or without isoproterenol or insulin for 1 h, as indicated. Aliquots of medium were taken to assay for glycerol content using Sigma glycerol reagent according to manufacturer's instructions. Cells were washed with PBS and lysed in 1% (w/v) Triton X-100 in PBS and assessed for protein concentration. Glycerol release was normalized to cellular protein content. Each sample was assayed in duplicate, and the average of the duplicates was considered as one biological replicate.

### [^3^H]Leucine incorporation assay for protein synthesis

3T3-L1 adipocytes in 24-well plates were washed twice and serum-starved in leucine-free DMEM (Sigma-Aldrich) supplemented with 0.2% BSA and 20 mm HEPES, pH 7.4, for 2 h with MitoPQ where indicated. [^3^H]Leucine (5 μCi/ml) (PerkinElmer Life Sciences) was added at the same time as the indicated doses of insulin for 1 h. To determine background leucine incorporation, 5 μm cycloheximide was added for 10 min before the addition of [^3^H]leucine. Leucine incorporation was terminated with three rapid washes in ice-cold PBS followed by incubating cells with ice-cold 10% TCA for 10 min to precipitate protein. Pellets were washed three times in ice-cold 10% TCA to remove free [^3^H]leucine. Pellets were resuspended in 50 mm NaOH with 1% Triton X-100 at 65 °C for 20 min. Samples were assessed for radioactivity, and results were normalized for protein content. Assays were performed in triplicate, and the average of the triplicate was considered as one biological replicate.

### General statistical analysis

Data are expressed as means ± S.E. Specified statistical tests were performed using GraphPad Prism version 6.01 for Windows (GraphPad Software). For data sets with multiple treatments, subgroups were initially compared with the relevant analysis of variance, depending on the levels of treatments. If row or column factors were significant, specific subgroups were then compared using Student's two-sample *t* test (adjusting for multiple comparisons using the Sidak method where indicated). One-sample *t* tests were used to test significance where described. Significant effects were defined as *p* < 0.05 by these *t* tests, as reported in the figures.

## Author contributions

D. J. F., M. P. M., and D. E. J. conceived of the study. D. J. F. and A. Y. M. performed the majority of experiments. K. C. T. aided with experiments. J. R. K. performed bioenergetics experiments. J. S. performed experiments in isolated soleus muscle. J. G. B. and D. J. H. performed live-cell microscopy experiments. S. T. C. and R. C. H. carried out chemical design and synthesis. G. J. M. and R. S. aided with measurement of superoxide and provided critical feedback on the project. D. J. F., A. Y. M., M. P. M., and D. E. J. wrote the paper. All authors reviewed the results, edited the manuscript, and approved the final version of the manuscript.

## Footnotes

5 The abbreviations used are:

ETC: electron transport chain
MitoPQ: mitochondria-targeted paraquat
MitoPQ-Ctrl: MitoPQ-control
PRDX: peroxiredoxin
*J*_O2_: rate of oxygen consumption
TNF: tumor necrosis factor
2DOG: 2-deoxyglucose
HFHSD: high-fat high-sucrose diet
JNK: c-Jun N-terminal kinase
PDGF: platelet-derived growth factor
PDGFR: PDGF receptor
AICAR: aminoimidazole-4-carboxamide ribonucleotide
AMPK: AMP-activated protein kinase
HA: hemagglutinin
DMEM: Dulbecco's modified Eagle's medium
KRP: Krebs–Ringer phosphate
RIPA: radioimmune precipitation assay
NEM: *N*-ethylmaleimide
MitoSOX: mito-hydroethidine
Mito-E^+^: mito-ethidium
Mito-2OHE^+^: mito-hydroxyethidium
CE: collision energy.
